# The Genetic Characteristics of FT-MIRS-Predicted Milk Fatty Acids in Chinese Holstein Cows

**DOI:** 10.3390/ani14192901

**Published:** 2024-10-08

**Authors:** Chunfang Li, Yikai Fan, Dongwei Wang, Chu Chu, Xiong Shen, Haitong Wang, Xuelu Luo, Liangkang Nan, Xiaoli Ren, Shaohu Chen, Qingxia Yan, Junqing Ni, Jianming Li, Yabin Ma, Shujun Zhang

**Affiliations:** 1 Key Laboratory of Agricultural Animal Genetics, Breeding and Reproduction of Ministry of Education, Huazhong Agricultural University, Wuhan 430070, China; chunfangli0521@webmail.hzau.edu.cn (C.L.); fanyikai123@webmail.hzau.edu.cn (Y.F.); wangdwei@webmail.hzau.edu.cn (D.W.); chu1999@webmail.hzau.edu.cn (C.C.); shenxx@webmail.hzau.edu.cn (X.S.); htw0411@webmail.hzau.edu.cn (H.W.); lxl775282323@163.com (X.L.); 15827557518@163.com (L.N.); renxl1990@163.com (X.R.); 2 Frontiers Science Center for Animal Breeding and Sustainable Production, Huazhong Agricultural University, Wuhan 430070, China; 3 Hebei Livestock Breeding Station, Shijiazhuang 050060, China; 13803337871@163.com (J.N.); li13785153452@163.com (J.L.); 4 Dairy Association of China, Beijing 100192, China; net@dac.org.cn (S.C.); 18601381139@wo.com.cn (Q.Y.)

**Keywords:** milk, Fourier Transform Mid-Infrared Spectroscopy (FT-MIRS), fatty acid content, genetic characteristics, genome-wide association study

## Abstract

**Simple Summary:**

Fourier Transform Infrared Spectroscopy (FT-MIRS) is widely used in milk quality detection, dairy herd improvement (DHI), and other fields. It is an economical, fast, accurate, and nondestructive batch tool for determining production performance phenotypes of dairy cows. Milk is one of the most important ways to provide the human body with the fatty acids it needs. There are a huge number of dairy cows in China. Therefore, it is possible to control fatty acid production in the milk source through targeted husbandry and breeding on large pastures to improve the quality of milk production. However, this work has not yet officially begun in China. In summary, our work uses FT-MIRS for the first time to study the phenotypic properties of milk fatty acid content and the genetic mechanism of its formation and to estimate genetic parameters. At the same time, SNPs significantly related to fatty acid content were discovered and the genes or adjacent genes had critical regulatory effects on milk fat synthesis, milk protein synthesis, adipocyte differentiation, mammary gland development, milk synthesis, and growth and development in dairy cows, thus providing a new perspective for cow genetic selection in China.

**Abstract:**

Fourier Transform Mid-Infrared Spectroscopy (FT-MIRS) can be used for quantitative detection of milk components. Here, milk samples of 458 Chinese Holstein cows from 11 provinces in China were collected and we established a total of 22 quantitative prediction models in milk fatty acids by FT-MIRS. The coefficient of determination of the validation set ranged from 0.59 (C18:0) to 0.76 (C4:0). The models were adopted to predict the milk fatty acids from 2138 cows and a new high-throughput computing software HiBLUP was employed to construct a multi-trait model to estimate and analyze genetic parameters in dairy cows. Finally, genome-wide association analysis was performed and seven novel SNPs significantly associated with fatty acid content were selected, investigated, and verified with the FarmCPU method, which stands for “Fixed and random model Circulating Probability Unification”. The findings of this study lay a foundation and offer technical support for the study of fatty acid trait breeding and the screening and grouping of characteristic dairy cows in China with rich, high-quality fatty acids. It is hoped that in the future, the method established in this study will be able to screen milk sources rich in high-quality fatty acids.

## 1. Introduction

Milk is a crucial source of human nutrition and essential to people’s lives and the agricultural economy [1]. Milk fat is a vital nutrient and also the primary source of essential fatty acids (linoleic acid and α-linolenic acid) for human beings [2,3,4]. Almost every kind of individual fatty acid has significant implications for human nutrition, health, and disease control [4,5,6].

Fatty acids can be divided into saturated fatty acids (about 70%) and unsaturated fatty acids (about 25% monounsaturated fatty acids and 5% polyunsaturated fatty acids) according to the chemical bonds of carbon chains [3,6,7]. They can also be classified into short-chain fatty acids, medium-chain fatty acids, and long-chain fatty acids according to the carbon chain length [8].

Supplementation and control of human consumption of fatty acids through milk boast essential biological and economic values in the future. Rapid batch examination of milk fatty acid content via genetic parameters accurately screens cows with high-yield high-quality fatty acids, presenting significant breeding value [1,3], and can also provide a reference for feed formula adjustment and healthy and efficient dairy cattle breeding [9].

Fourier Transform Mid-Infrared Spectroscopy (FT-MIRS) can analyze the frequencies and peaks absorbed by specific chemical bonds of molecules in milk substances, which can be utilized to identify the properties or content of milk substances [10,11,12]. FT-MIRS has been extensively utilized in milk composition detection and global dairy herd improvement (DHI) due to its low-cost, simple, rapid, batch-testing, accurate, and nondestructive nature [13,14].

According to the Beer–Lambert law, FT-MIRS absorbance values are linearly correlated with relevant milk components [15]. Thus, as the most widely used machine learning algorithm, partial least squares regression (PLSR) was employed to establish a quantitative prediction model based on FT-MIRS, especially in the establishment of human consumption fatty acid prediction models [9,10,16,17,18]. Before model training, spectral data should be preprocessed, and essential waves should be selected. Preprocessing is the step of performing various transformations and processing of raw spectral data to improve the accuracy and robustness of the model and to reduce noise and interference for better extraction of useful information. Common preprocessing algorithms include Savitzky–Golay (SG) [19], multivariate scatter correction (MSC) [20,21], standard normal variable transformation (SNV) [22], first derivative (D1) [21,23], second derivative (D2) [3], etc. [24]. Moreover, it was revealed that random forest regression (RFR), support vector machine regression (SVR), and ridge regression (RR) algorithms could also construct quantitative prediction models for milk composition [25,26,27].

Fatty acids are a quantitative and complex trait modulated by multiple factors [28], including genetics, physiological properties (breed, parity, stage of lactation, and calving season), and rearing environment [10,29,30,31,32,33,34,35]. In particular, the diet of dairy cows has a significant impact on the fatty acid composition of milk [30,32]. There are some phenotypic and genetic correlations among various human consumption fatty acids and between fatty acids and milk production traits [36,37]. In addition, it was suggested that FT-MIRS waves were heritable and related to the genetic characteristics of relevant milk components [11,38,39,40,41,42]. These results provided a new research idea for selecting high-yield and high-quality fatty acid cows.

Genome-wide association study (GWAS) is a prominent method for analyzing genetic variations of complex traits. There have been numerous studies concerning human/animal/plant genes based on GWAS. Regarding dairy cattle, GWAS has been applied to study milk production, reproduction, and health for years [43,44]. Multiple Single Nucleotide Polymorphism (SNP) chips have been developed to assist detection [45]. 50k, 100k, and 777k SNP chips based on UMD_3.1 have been broadly applied [22,44,46,47,48,49,50,51,52,53,54], while studies based on ARS_UCD 1.2 are fewer [55]. Moreover, the GWAS model based on multiple types of information directly affects the accuracy of SNP screening [51,56]. Currently, models extensively employed in research are the General Linear Model (GLM) and Mixed Linear Model (MLM) based on pedigree and SNP chip information [57,58]. Hence, constructing new models and algorithms for various fields or species is significant to GWAS application development.

Fatty acid traits have medium–high heritability and are controlled by multiple genes [54,59,60,61,62,63], such as the *DGAT1*, which regulates milk fat synthesis [51]. However, genes regulating fatty acid content in milk and their SNPs remain to be elucidated. GWAS can effectively screen significant SNPs and genes substantially affecting fatty acid content [22,43,51,64].

This study intended to build quantitative models to predict milk fatty acid content in a large sample of Chinese Holstein cows using FT-MIRS. Then, a fatty acid multi-trait model was constructed to evaluate the fatty-acid breeding value, heritability, repeatability, phenotypic and genetic correlations, etc. Eventually, phenotypic indices of fatty acid content predictions were processed for GWAS to screen SNPs significantly associated with fatty acid content. Validation was carried out in an independent herd to further identify fatty acid content and critical SNPs. This study delves into phenotypic traits of milk fatty acid content and its genetic mechanism. It provides new technologies and insights for selecting cows that produce milk rich in high-quality fatty acids and establishing a core cattle herd.

## 2. Materials and Methods

### 2.1. Milk Sample Collection, FT-MIRS, and Fatty Acid Content Reference Value Measurement

The Animal Management and Ethics Committee of Huazhong Agricultural University reviewed and approved the experimental protocol for this project (HZAUCA-2019-004). The feeding and management conditions of all cows were similar to previous studies [65]. All cows were reared in large pens with the total mixed ration (TMR) feeding mode (twice a day) with ad libitum access to water and free milking. The TMR mainly contains the following feeds: roughage such as corn silage and alfalfa hay; concentrated feed such as corn, soybean meal, and barley; and vitamins, minerals, and feed additives. The average ambient temperature was 8 °C–27 °C, and the relative humidity was 45–75%.

Milk samples were collected from 458 Chinese Holstein cows from 36 dairy farms in 11 provinces of China from December 2019 to May 2021 (Table S1). According to the NY/T 1450-2007 technical specification for the performance measurement of Chinese Holstein cattle, from each cow was harvested one milk sample via an automatic milking device. Each milk sample (40 mL) was collected and added into new cylindrical bottles (diameter 3.5 cm, height 9 cm). The bottles were numbered in order. Bronopol preservative (0.35 μL) was immediately added to each sampling bottle and slowly shaken to dissolve it thoroughly. Ice bags (2–4 °C) were placed around the milk samples to prevent spoilage during transportation back to the laboratory.

In order to ensure the results’ reliability, all experiments were carried out on fixed instruments in the same laboratory, and the instruments were standardized regularly. After arriving at the laboratory, samples were placed in the water bath at 42 °C for 15–20 min. FT + (Foss, Hillerød, Denmark) milk composition detector was utilized, and the solid fiber probe was dipped into the liquid, mixing, and scanning samples to generate FT-MIRS data. The data consisted of 1060 waves in the wavenumber interval from 925.66 cm^−1^–5010.15 cm^−1^, covering two noise-like regions (1597.21 cm^−1^–1712.95 cm^−1^, 3063.25 cm^−1^–3641.95 cm^−1^); the other areas were fingerprint regions. The reference value of human consumption fatty acids was determined using gas chromatography (GC) of Hou et al. [66]. Lipids were extracted with chloroform/methanol extraction from milk samples before the determination, and then the fatty acids were esterified with a KOH methanol solution. Finally, the supernatant was filtered with a membrane and uploaded to the device. In total, 37 individual fatty acids were determined, expressed as g/100 g total fat.

Milk samples were screened by the following criteria: milk fat percentage 1.5–9%; milk protein percentage 1–7%; somatic cell counts ≤ 1,000,000/mL; the relative error of two repeated measurements of the same fatty acid in the same sample not be greater than 10%; and FT-MIRS standard Mahalanobis distance (**GH**) ≤ 3, calculated by the following formula:

$$GH(x,y)=\sqrt{{\left(x-y\right)}^{T}S^{-1}(x-y)}$$

where **x** and **y** denote two random samples, and **S** is the covariance matrix. A total of 359 milk samples were selected for the subsequent prediction model.

### 2.2. Fatty Acid Content Prediction Model Construction

According to GC results, 14 individual fatty acids and 8 fatty acid groups were selected to establish the prediction model, and the composition of fatty acid groups is shown in Table 1 [54]. For each type of fatty acid, 75% of samples were randomly chosen for model training and random 10-fold cross-validation, and 25% for model testing. For each prediction model, we separated the windows according to the spectral absorption region characteristics of different chemical functional groups, and then manually added or subtracted the critical wavelengths of each window; in the end, 459–709 characteristic waves were selected. The spectrum of water expression was not deleted when selecting characteristic waves because some studies showed that the absorbance of human consumption fatty acid-related chemical functional groups might be related to water absorbance [39,67,68,69,70]. FT-MIRS data were preprocessed twice before the model establishment, with first derivative (D1) or second derivative (D2) employed [71]. Studies have demonstrated that the D1 and D2 preprocessing methods contribute to enhancing the signal and extracting spectral points relevant to milk fatty acids. The model was trained using the PLSR algorithm, and the principal components of PCA were 8–10. The development environment was Jupyter Notebook version 6.5.4 based on Python version 3.8.

The coefficient of determination (R_cv_^2^) and root mean square error (RMSE_cv_) between the cross-validation set and the predicted phenotypes, the coefficient of determination (R_t_^2^) and root mean square error (RMSE_t_) between the test set and the predicted phenotypes, and the residual prediction bias (RPD_cv_) of the cross-validation set were used to evaluate the performance of the prediction models. The optimal model was obtained for each sort of fatty acid. All the above evaluation metrics were calculated using the SciPy package in Python version 3.8; the formulas are as follows:

$$\begin{array}{l} RMSE=\sqrt{\frac{\sum_{n=1}^{N}\left[{\left(y_{n}-{\hat{y}}_{n}\right)}^{2}\right]}{N}}\\ R^{2}=1-\frac{\sum_{n=1}^{N}{\left(y_{n}-{\hat{y}}_{n}\right)}^{2}}{\sum_{n=1}^{N}{\left(y_{n}-\overset{‾}{y}\right)}^{2}} \end{array}$$

$$RPD=\frac{{STD}_{EV}}{RMSE}$$

where $y_{n}$ and ${\hat{y}}_{n}$ represent the reference value and predicted value. $\bar{y}$ is the mean of the y values, **N** represents the sample size, and **STD_EV_** represents the standard deviation of the sample.

### 2.3. Fatty Acid Content Prediction and Correction

Considering the samples from North China accounted for the most significant proportion in the prediction model with about 1/3 of the total samples, 2138 Chinese Holstein cows were selected from five cattle farms in North China. Corresponding pedigree data were collected and statistically analyzed. The prediction models based on 14 individual fatty acids and 8 fatty acid groups predicted 22 fatty acids in 12,236 milk samples from 2138 cows. Corrected phenotypic values of 22 fatty acids were obtained using the general linear mixed model established by the R (V4.2.1) package lmertest. The corrected formula is as follows:

$$Y_{ijkm}=\mu +P_{i}+L_{j}+S_{k}+{HTD}_{m}+e_{ijkm}$$

where **μ** stands for the mean value of the predicted traits; **P_i_** represents parity, which is divided into 5 levels (i = 1, 2, 3, 4 and 5–10); **L_j_** denotes the lactation period, ranging from 5 to 365 days, divided into 12 levels with 30 days as a unit (j = A–L); **S_k_** stands for calving season effect, which is divided into 4 levels (k = Spring, Summer, Autumn, and Winter); **HTD_m_** represents herd-test-date random effects (the date format: DD/MM/YY); **e_ijklm_** is the random residual effect. As for GWAS, the corrected phenotype was the mean of all predicted values per cow minus associated fixed effects (Folder S1).

### 2.4. Genetic Parameter Evaluation

In this study, the multi-trait animal model in HiBLUP v1.5.0 (a new high-throughput computing software) was used to construct the relationship matrix (HRM) based on pedigree and genome data [72]. This was the case so that the influence of the “bull factor” would already be included in the model, which takes into account the combined effects of pedigree and genotype. This approach (of not including the bull factor when using HiBLUP) has been already used to capture genetic variance in high-throughput genomic studies. There are 2578 individual animals included in the pedigree. The variance components of fatty acid traits were estimated using the SSGBLUP model with the AIREML algorithm [73,74], and the heritability, repeatability, and phenotypic and genetic correlations of the traits were calculated based on the variance. Moreover, the individual breeding value was estimated. The accuracy of breeding value estimation was assessed by the correlation coefficient between the phenotype and individual breeding value after model correction. Because the phenotypic and genetic correlations between 22 fatty acids and 5 conventional milk components (milk fat, milk protein, lactose, total solids, and urea nitrogen, measured by FOSS milk component analyzer) were to be investigated in the process of genetic parameter estimation, 27 traits are in the multi-model analysis. Taking the predicted values of two traits as an example, the model is defined as follows:

$$\left[\begin{array}{c} y_{1}\\ y_{2} \end{array}\right]=\left[\begin{array}{cc} X_{1} & 0\\ 0 & X_{2} \end{array}\right]\left[\begin{array}{c} b_{1}\\ b_{2} \end{array}\right]+\left[\begin{array}{cc} Z_{1} & 0\\ 0 & Z_{2} \end{array}\right]\left[\begin{array}{c} u_{1}\\ u_{2} \end{array}\right]+\left[\begin{array}{cc} W_{1} & 0\\ 0 & W_{2} \end{array}\right]\left[\begin{array}{c} c_{1}\\ c_{2} \end{array}\right]+\left[\begin{array}{c} e_{1}\\ e_{2} \end{array}\right]$$

In the model, **y_1_** and **y_2_** represent the vectors of predicted trait values; and **b_1_** and **b_2_** stand for vectors of fixed effects, including the parity effect and lactation effect. Parity is divided into 5 levels (1, 2, 3, 4, and 5–10) and lactation (5 to 365 days) is divided into 12 levels with 30 days. **u_1_** and **u_2_** are vectors of individual additive genetic effects, which distributes in u-N (0, $\sigma_{u}^{2}$). **c_1_** and **c_2_** are individual permanent environmental effects vectors, which accord with the distribution c-N (0,$\sigma_{c}^{2}$). **e_1_** and **e_2_** are residual effect vectors, consistent with the distribution e-N (0,$\sigma_{e}^{2}$). **X_1_**, **X_2_**, **Z_1_**, **Z_2_**, **W_1_**, and **W_2_** are the structure matrices of fixed effects, individual additive genetic effects, and permanent environmental effects, respectively. The maximum number of iterations of the AIREML algorithm is 300 and the convergence is assessed by the formula:

$$\sqrt{\frac{\Sigma {\left(\beta^{t}-\beta^{t-1}\right)}^{2}}{\Sigma {\left(\beta^{t}\right)}^{2}}}<1\times 10^{-6}$$

where ***β*** is the vector of all variances and covariances to be estimated, and ***t*** is the number of iterations.

In the estimation of model variance components, $\sigma_{p}^{2}$ denotes the total variance of the predicted value, $\sigma_{u}^{2}$ is the additive genetic variance, $\sigma_{c}^{2}$ describes the permanent environmental variance, and $\sigma_{e}^{2}$ represents the residual variance. Thus, $\sigma_{p}^{2}\;$=$\sigma_{u}^{2}\;$+$\;\sigma_{c}^{2}\;$+$\sigma_{e}^{2}$. The formulas for heritability and repeatability are h^2^ =$\frac{\sigma_{u}^{2}}{{{\sigma_{u}^{2}+\sigma }_{c}^{2}+\sigma }_{e}^{2}}$ and t = $\frac{\sigma_{u}^{2}+\sigma_{c}^{2}}{{{\sigma_{u}^{2}+\sigma }_{c}^{2}+\sigma }_{e}^{2}}$, respectively, and the formulas for genetic and phenotypic correlations are r_G_ =$\frac{{Cov}_{u_{1}u_{2}}}{\sigma_{u_{1}}\sigma_{u_{2}}}$ and r_P_ =$\frac{{Cov}_{p_{1}p_{2}}}{\sigma_{p_{1}}\sigma_{p_{2}}}$.

### 2.5. GWAS and Screening of Fatty Acid-Related SNPs

In this study, Illumina Bovine SNP 50k (47873 SNPs, genome version ARS_UCD 1.2) was applied to detect the genotypes of 1873 Chinese Holstein cows. The selected cows were healthy, with consistent feeding and management conditions and no illness within half a year. The parity was 1–10, the lactation period was 5–365 d, and the calving season was evenly distributed in spring, summer, autumn, and winter. GWAS was performed to preliminarily screen significant SNPs based on the corrected fatty acid content of 1873 Chinese Holstein cows. 50k chip data quality control was performed before GWAS. A total of 30 chromosomes were selected for SNP analysis, including 29 autosomes (numbered 1–29) and 1 sex chromosome (X). A total of 47,873 SNP genotypes of 1873 dairy cows were analyzed by Plink 1.9 software [75]. The SNP loci and individual cows with a genotype detection rate greater than 0.98 and SNPs with a Hardy–Weinberg equilibrium of less than 0.0001 were screened out. Finally, the remaining 1712 cows and 42,500 SNP were obtained for fatty acid GWAS.

The analysis tool was the rMVP package in the R language [76]. The relationship matrix was constructed using pedigree data, phenotypic data, and SNP markers. In order to control the impact of population structure and kinship on GWAS and reduce the generation of false positives, three models were used in rMVP: the general linear model (GLM), mixed linear model (MLM), and FarmCPU were adopted to analyze the relationship matrix. The GLM used principal component analysis; the formula can be presented as y = PCs + S + e, where y represents the phenotype, PCs represents the principal components of the population structure, S represents the gene markers, and e represents the residual [77]. The MLM adds kinship (K) as a covariate to improve the test power of the model (y = PCs + S + K + e) [78], while FarmCPU eliminates the confounding between PCs and K, allowing fixed effect and random effect models to be executed independently [79]. The value of PCs in this study was determined to be 5. SNPs significantly associated with fatty acid content were preliminarily selected based on three models above. The threshold for acceptance in GWAS is approximately $1\times 10^{-6}$, calculated via $\frac{0.05(alpha\;value)}{42500(SNP\;maker\;size)}$. We use the gene-based module in ANNOVAR to annotate candidate genes corresponding to SNPs [80].

Based on the preliminarily selected SNPs and their DNA sequences, the SNPs of 265 Chinese Holstein cows from an independent herd were sequenced using the sequencing technology, and the SNP genotypes of 265 Chinese Holstein cows were obtained. The association between SNP and fatty acid content was analyzed by Bonferroni ANOVA with an alpha value of 0.05. Each SNP was compared on 3 genotypes (AA, AB, and BB) and important SNPs significantly correlated with fatty acid were further verified and identified.

## 3. Results

### 3.1. Milk Fatty Acid Content Trait Analysis

Table 2 shows the fat reference values in two units (g/100 g of total fat, percentage of total fatty acid). The average content of individual fatty acid (in units of g/100 g total fat) was 0.10 (C18:3,n3cis-9,12,15)–12.74 (C16:0). The coefficient of variation (CV) was 48.60% (C16:0)–90.55% (C4:0). The mean value of fatty acid groups was 1.33 (PUFA)–35.22 (TFA), and the CV was 48.55% (SFA)–58.07% (SCFA). The mean values of individual fatty acids in the percentage of total fatty acids ranged from 0.26 (C18:3,n3cis-9,12,15) to 36.37 (C16:0), and the coefficients of variation ranged from 9.69% (C16:0) to 92.01% (C4:0). The average content of the fatty acid groups ranged from 3.71 (PUFA) to 72.30 (SFA) with coefficients of variation ranging from 6.23% (SFA) to 37.76% (SCFA). The results indicate a wide range of milk fatty acid content of dairy cows from 36 dairy farms in 11 provinces in China; therefore, this dataset is suitable for model establishment.

### 3.2. FT-MIRS-Based Fatty Acid Prediction Model

**Figure 1:**
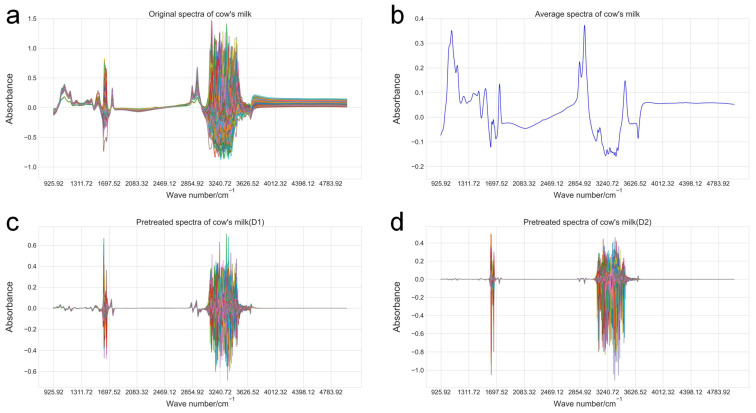
The spectra of cow’s milk(lines of different colors represent different samples). (**a**) The original spectra of all reference samples. (**b**) The average spectra of all reference samples. (**c**) The D1 preprocessing spectra of all reference samples. (**d**) The D2 preprocessing pretreated spectra of all reference samples.

Figure 1 illustrates the FT-MIRS absorbances of all milk samples for prediction model development. Figure 1a delineates raw spectra, and Figure 1b represents averaged spectra. All prediction models in this study were based on the first derivative (D1) or second derivative (D2) FT-MIRS data preprocessing methods, and preprocessed spectra are shown in Figure 1c,d. Table 3 summarizes the relevant information and model evaluation indicators of the optimal prediction models for 22 fatty acids, including preprocessing methods, number of characteristic waves, R_cv_^2^, RMSE_cv_, R_t_^2^, RMSE_t_, and RPD_cv_.

The model preprocessing method presents a certain regularity: among individual fatty acids, D1 was used more frequently, while D2 was used uniformly in fatty acid groups, suggesting that D1 was more suitable for individual fatty acids with low content than D2. R_cv_^2^ ranged from 0.59 (C18:0) to 0.76 (C4:0), and R_t_^2^ ranged from 0.44 (C18:3,n3 cis-9, 12, 15) to 0.74 (SCFA). Among individual fatty acids, R_cv_^2^ and R_t_^2^ were apt to decrease with the carbon chain lengthening, and Rt^2^ exhibited more significant variation. The R_cv_^2^ of single unsaturated fatty acid is higher than that of saturated fatty acid in the same carbon chain. Among fatty acid groups, except MUFA and PUFA, R_cv_^2^ and R_t_^2^ manifested congruous regularity, SFA > TFA > UFA, SCFA > MCFA > LCFA, which further indicated that model performance was negatively correlated with carbon chain length. In addition, it was found that the overall levels of R_cv_^2^ and R_t_^2^ of fatty acid groups were slightly higher than those of single fatty acids, suggesting that fatty acid content and carbon chain length could influence model performance. The RPD_cv_ ranged from 1.57 (C18:0) to 2.04 (C4:0), indicating that the model performed acceptably and could predict fatty acid content and be used in genetic parameter estimation and GWAS for subsequent dairy herds.

### 3.3. Genetic Parameter Estimates of Fatty Acid Content

#### 3.3.1. Heritability, Repeatability, and Individual Breeding Value

Table 4 describes the estimates of variance components, heritability, repeatability, and individual breeding values of 22 fatty acids. Except for C17:0, all 22 fatty acids had medium heritability and medium to high repeatability. Among individual fatty acids, C12:0 (0.34) had the highest heritability, and C17:0(0.20) had the lowest. SFA (0.31) exhibited the highest heritability among fatty acid groups, and MCFA (0.27) showed the lowest. The heritability of individual fatty acids varied substantially, while fatty acid groups had similar heritability with a high overall level, consistent with repeatability. This indicates that the differentiating features of single fatty acids were more prominent, while those of fatty acid groups might be weakened by containing multiple fatty acids.

Regarding the individual breeding value of dairy cows, the value of fatty acids varied widely and the accuracy was estimated to be high (0.54–0.82). In total, 15 of the 22 fatty acids had accuracy above 0.7, denoting that the individual breeding value estimation method in this study could be used to screen dairy cows with high or low fatty acid content and could improve the breeding efficiency of fatty acid production traits.

#### 3.3.2. Genetic and Phenotypic Correlations

Table 5 illustrates the estimates of genetic and phenotypic correlations among 27 traits (22 fatty acids and 5 conventional milk components), and the two correlations had similar trends. For further description of genetic findings, see Table 5:

Correlations between fatty acids: Except for C4:0 and C14:1, cis-9 (−0.01), other fatty acids were positively associated (0.04–1.00). And correlations between individual fatty acids and fatty acid groups were medium to high. The heritability of almost all fatty acid groups except LCFA was above 0.6. Correlations between individual fatty acids and fatty acid groups were primarily low to moderate, and C8:0, C16:0, C17:0, and C18:0 were moderately/highly associated with fatty acid groups. Furthermore, fatty acids with similar carbon chain length or similar chemical functional groups had generally high correlations, and the correlation between single unsaturated fatty acids, UFA, MUFA, PUFA, and saturated fatty acids or other fatty acid groups was generally low. Overall, genetic correlation among fatty acids had an evident regularity, involving carbon chain length, chemical functional group type, and saturation degree, which could be used as a reference in the breeding process of fatty acid production traits.

### 3.4. Screening and Identification of Fatty Acid-Related SNPs and Genes

#### 3.4.1. Comparison and Filtering of the Three GWAS Methods

**Figure 2:**
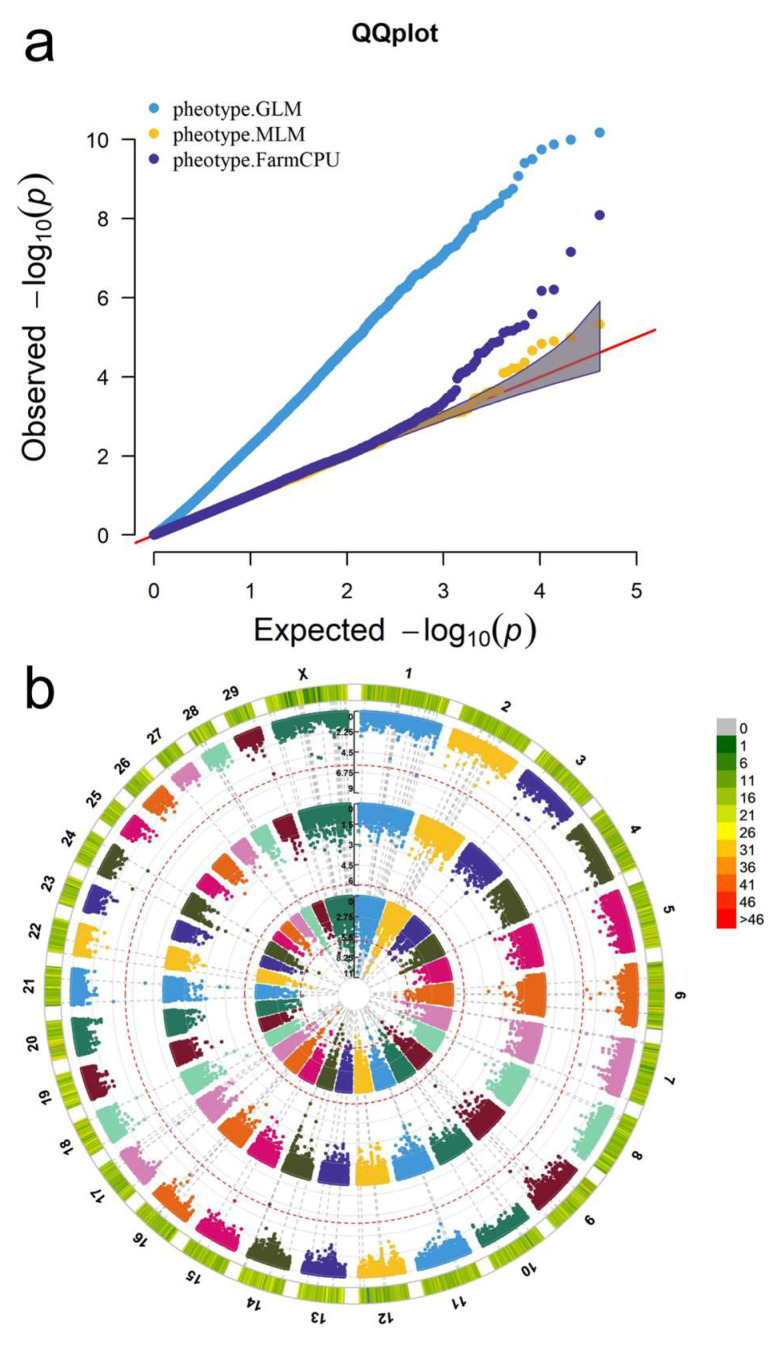
Multraits-QQplot and Circular Manhattan for C6:0. (**a**) The quantile–quantile plot based on the GLM, MLM, and FarmCPU methods. The red line represents x = y. The grey shaded place represents the 0.95 confidence interval (**b**) The cyclic Manhattan plot based on the GLM, MLM and FarmCPU methods combined with chip density.

The GLM, MLM, and FarmCPU methods were applied to the GWAS of 22 fatty acid content, as shown in the quantile–quantile plot (Figure 2a). The distribution of 42,500 SNPs on 30 chromosomes is shown in Figure 2b. Taking C6:0 as an example (the results of other fatty acids were similar, see the link for details: https://github.com/YikaiFan0908/FA (accessed on 3 July 2024)), Figure 2a demonstrated many false positives among selected significant SNPs via the GLM. The MLM significantly improved on false positive control but did not select significant SNPs. FarmCPU combines the advantages of the first two methods, which can control false positives and have more robust SNP screening performance. Thus, the FarmCPU method was selected for GWAS and validation.

#### 3.4.2. GWAS, Screening, and Identification of Fatty Acid Content Associated SNPs

Based on the new version of the ARS_UCD 1.2 genome, SNP genotypes of 1873 Chinese Holstein cows were detected by 50k SNP microarray. Then, SNPs associated with fatty acid content were identified by the FarmCPU model. Table 6 exhibits SNP data and validation of preliminarily selected SNPs. In this study, 39 SNPs screened from 20 chromosomes were prominently associated with 17 fatty acids (13 single fatty acids and 4 fatty acid groups), of which 8 SNPs were selected twice. Hence, the total number of SNPs was 47. Of the 17 fatty acids, 14 were significantly associated with 2–5 SNPs each. Five SNPs were correlated with C8:0, C14:1, and cis-9, and four SNPs were associated with C6:0 and C10:0. A total of 37 of the 47 SNPs were matched with corresponding genomes from reference genomes.

**Figure 3:**
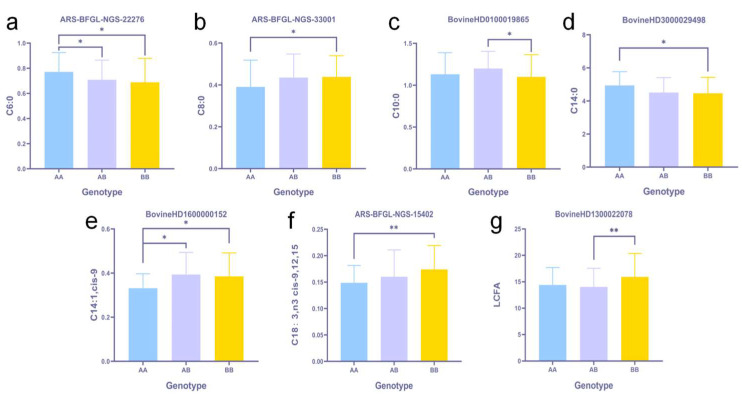
Seven validated SNPs significantly related to fatty acid content.‘*’ means *p*-Value Bonferroni < 0.05, and ‘**’ means *p*-Value Bonferroni < 0.01. (**a**) Difference and significance of C6:0 among three genotypes of ARS-BFGL-NGS-22276 gene; (**b**) Difference and significance of C8:0 among three genotypes of ARS-BFGL-NGS-33001 gene; (**c**) Difference and significance of C10:0 among three genotypes of Bovine HD 0100019865 gene; (**d**) Difference and significance of C14:0 among three genotypes of Bovine HD 3000029498 gene; (**e**) Difference and significance of C14:1,cis-9 among three genotypes of Bovine HD 1600000152 gene; (**f**) Difference and significance of C18:3,n3 cis-9/12/15 among three genotypes of ARS-BFGL-NGS-15402 gene; (**g**) Difference and significance of LCFA among three genotypes of ARS-BFGL-NGS-15402 gene.

The analysis of the effect of 3 SNPs on the content of short-chain fatty acids showed the following: The C6:0 content of AA genotype cows with SNP (ARS-BFGL-NGS-22276) was significantly higher than that of AB and BB genotypes, showing AA > AB > BB, indicating that allele A has an up-regulation effect on C6:0 expression compared with B (Figure 3a). The AA genotype of SNP (ARS-BFGL-NGS-33001) had significantly lower C8:0 content than the BB genotype, denoting BB > AB > AA, indicating that the allele A had a down-regulation effect on C8:0 expression compared with the allele B (Figure 3b). The AB genotype of SNP (Bovine HD 0100019865) had significantly higher C10:0 content than the BB genotype, showing AB > AA > BB, suggesting that heterozygotes may have an up-regulation effect on C10:0 expression compared with homozygotes (Figure 3c).

Individual medium-chain fatty acids: 1, 2, 5, 3, and 3 SNPs were found to be significantly associated with C12:0, C14:0, C14:1, cis-9, C15:0, and C16:0, respectively. The 14 SNPs were distributed on 8 chromosomes, 7 of which were located in introns, 3 in intergenic regions, and 4 were not associated with corresponding genes. C15:0 and C16:0 had identical SNPs, BovineHD0400022351, BovineHD2200010872, and BovineHD3000019134, which were located in an unknown gene region, the intron of *SYNPR*, and the intron region of *TRPC5*. After ANOVA with the Bonferroni test, two SNPs were identified from 14 SNPs, Bovine HD 3000029498 (C14: 0, *p*-value Bonferroni = $3.56\times 10^{-2}$) and Bovine HD 1600000152 (C14:1,cis-9, *p*-value Bonferroni = $1.57\times 10^{-2}$), which were located between *CASK* (dist = 28,036) and *NYX* (dist = 6763) and between *ENSBTAG00000050274* (dist = 75,663) and *ADORA1*(dist = 18,476), respectively.

Analysis of the effect of the two SNPs on the content of medium-chain fatty acids showed the following: The C14:0 content of AA genotype cows with SNP (Bovine HD 3000029498) was significantly higher than that of AB and BB genotypes, showing AA > AB > BB, indicating that allele A has an up-regulation effect on C14:0 expression compared with B (Figure 3d). The AA genotype of SNP (Bovine HD 1600000152) had significantly lower C14:1,cis-9 content than the AB and BB genotypes, denoting AB > BB > AA, indicating that homozygotes may result in the down-regulation of C14:1, cis-9 expression compared with heterozygotes (Figure 3e).

Individual long-chain fatty acids: 2, 3, 3, and 3 SNPs were found to be significantly associated with long-chain fatty acids C18:0, C18:1,n9 cis-9, C18:2,n6 cis-9/12, C18:3,n3 cis-9/12/15, respectively. These 11 SNPs were distributed on 9 chromosomes. Among them, 5 were located within intergenic regions, 2 in introns, 1 in exon, and 3 were unrelated to corresponding genes. Via ANOVA with the Bonferroni test, ARS-BFGL-NGS-15402 was found to have a considerable effect on C18:3, n3 cis-9/12/15 (*p*-value Bonferroni = $8.34\times 10^{-3}$) and was located in the intron of *TTC29*.

The post-validation effect analysis for the SNP showed the following: The C18:3, n3 cis-9/12/15 of cows with the AA genotype was significantly lower than that of cows with the BB genotype, and BB > AB > AA, manifesting that allele A had a down-regulation effect compared with allele B (Figure 3f).

Fatty acid groups: 1, 2, 3, and 3 SNPs were found to be significantly correlated with UFA, MUFA, SCFA, and LCFA by 50k SNP microarray, respectively. The 7 SNPs were distributed on 5 chromosomes, 4 located in introns, and 3 were not associated with corresponding genes. ARS-BFGL-NGS-27018 was significantly relevant to UFA and MUFA. After ANOVA with the Bonferroni test, Bovine HD 1300022078 was confirmed to have a remarkable effect on LCFA (*p*-value Bonferroni = $4.61\times 10^{-3}$) and was located within the intron of *EYA2*. The post-validation effect analysis revealed that the LCFA content of AB genotype cows was significantly lower than that of BB genotype cows, and BB > AA > AB, indicating that heterozygotes may have a down-regulation effect compared with homozygotes (Figure 3g).

## 4. Discussion

### 4.1. Reference Value of Fatty Acid Measurements and Prediction Model Construction

Individual fatty acids in milk are difficult to determine due to their low concentration. Measurements from various studies vary globally [9,10,21,81,82]. The reference values in this study were collected from 36 large-scale cattle farms with more than 1000 cows. Given China’s enormous population and copious milk demand, massive numbers of dairy cows are reared by large-scale cattle farms in large pens with the TMR feeding mode. Only minor cattle farms and individual farmers use grazing systems. Thus, reference value measurements in this study differ from those in countries where dairy cows graze. Even though the reference values of each fatty acid essentially conformed to the normal distribution, the overall CV for g/100 g total fat was high, with an average of 57.31%. Yet, the average CV for the percentage of total fatty acid was reduced to 26.02% and more consistent with other studies [24]. This suggests that the samples were diverse and representative and applied to the model establishment [83]. A larger variation will improve the generalization ability of the prediction model, and g/100 g total fat can be used to calculate the absolute content of fatty acids in milk, which is more valuable for evaluating cow production performance and milk quality.

This study established 22 fatty acid prediction models based on the most widely used PLSR algorithm, and only D1 or D2 was performed for spectral preprocessing. The purpose was to reduce the differentiation between prediction models during prediction caused by distinct preprocessing or machine learning algorithms, which would affect genetic parameter estimates or GWAS. Similar to the results of Soyeurt et al., R_cv_^2^ and R_t_^2^ both kept high levels [9,10,16,18,84,85], and it was found that the performance of the superior model was related to carbon chain length, saturation degree, and substance content. The model developed in this study has poor predictive ability for some of the LCFA, e.g., C18:0, C7:0. The main reason for this is the low content of LCFA and the high proportion of unsaturated fatty acids in LCFA, which are relatively chemically unstable [86]. For example, Tiplady et al. also reported that R_cv_^2^ of SCFA, MCFA, and LCFA decreased with the lengthening of carbon chains [22]. Meanwhile, Soyeurt et al. revealed that saturated fatty acids performed better than unsaturated fatty acids [3]. Compared to Zhao’s study, ours involved 36 ranches in 11 provinces, with a wider generalization capacity and application of the model [87]. Thus, the model established in this study was superior and represented the characteristics of Chinese Holstein cows, which could be used to predict the fatty acid content of Chinese Holstein cows and analyze their genetic characteristics.

### 4.2. Fatty Acid Genetic Parameter Estimates and Comparison Analysis

Improving milk composition and quality-related traits via FT-MIRS is a general trend [11]. In this study, we used HiBLUP (https://www.hiblup.com/ (accessed on 3 July 2024)), a high-performance computing software developed by our laboratory, to estimate the genetic parameters of dairy cows for the first time. The HRM was constructed by making full use of genome and pedigree information and converged in a finite number of iterations. This study demonstrated moderate heritability, similar to Freitas et al. study. SFA (h^2^ = 0.305) showed the highest heritability among fatty acid groups [52,54,83]. Our repeatability estimates were higher than in previous studies. Tiplady et al., Lopez-Villalobos et al. and other studies reported low to medium repeatability of fatty acids with universally high breeding accuracy [22,37]. This indicated that our genetic analysis method and the predictions using FT-MIRS models could preliminarily screen dairy cows with high or low fatty acid content.

Furthermore, similar to the results of Lopez-Villalobos et al., phenotypic and genetic correlation exhibited identical trends. Milk protein was negatively associated with unsaturated fatty acids, while it was positively correlated with saturated fatty acids genetically. Lactose was negatively correlated with most fatty acids [37]. Similar to the study of Eskildesn C.E et al., there were high correlations between milk fat and fatty acids, and between different fatty acids in this study, which was affected by factors such as breed and feed [85]. Unlike previous studies, the correlations in this study were almost all positive. The reason might be that several models did not have high accuracy among the 22 fatty acid prediction models, leading to deviation. Also, this was possibly due to the low fatty acid production in the selected dairy herd with g/100 g of total fat as the unit, resulting in insignificant interaction between different fatty acids, suggesting room for breeding improvement. Much published work on milk fatty acids has found that correlations with FTIR data are improved when the fatty acid data are reported on the basis of g/dl milk instead of g/100 g fat [11,83]. Given overall positive correlations among fatty acids, we recommend selecting cows with high total fatty acid production, assuring high levels of single fatty acid and fatty acid groups. Subsequently, characteristic cows rich in a certain fatty acid can be selected according to genetic correlation and phenotypic correlation to establish the characteristic core group of dairy cows.

### 4.3. Fatty Acid-Related Molecular Markers and Corresponding Genes

Most existing research on fatty acid GWAS and SNP screening is based on the reference values determined by GC or other chemical methods [52,83,88]. Accurate fatty acid phenotype results can contribute to efficient SNP marker mining. However, determining sample reference values is time-consuming with a high cost, unable to obtain a large number of fatty acid phenotypes in a short time or to perform GWAS, breeding value estimation, and high-yield dairy cow selection in large herds. Nevertheless, GWAS based on the predicted values of fatty acid models established with FT-MIRS can effectively solve this problem, and the accuracy of the prediction model is required. In recent years, there are also reports on GWAS of fatty acids based on model predictions [51,54]. Tiplady et al. compared fatty acid GWAS results from direct measurement and predicted by the FT-MIRS model, showing that SNPs screened by the two methods were partially the same. This revealed that FT-MIRS model predictions have the potential to be used as alternative reference values to investigate genetic variation in fatty acids [22]. Thus far, there are no reports on GWAS using FT-MIRS model prediction in Chinese Holstein cows.

Researchers have previously conducted GWAS on the fatty acid profile in milk, and most studies have found that the two genes *DGAT1* and *SCD* are significantly associated with milk fatty acids based on UMD_3.1 [22,24,43,49,51,88]. In addition, Tiplady et al. found that *CCDC57* and *GPAT4* may be important fatty acid-related candidate genes [22]; Cruz et al. considered *PLBD1* and *MGST1* to be important additional candidate genes in Holstein cattle [51]; Buitenhuis et al. proposed that *ACSS3* is a dominant candidate gene for the QTL of C10:0 and C15:0 on chromosome 5 [88]; Li et al. identified 20 novel promising fatty acid candidate genes including *HTR1B*, *CPM*, and *PRKG1* [24]. In an attempt to explore whether novel SNPs significantly associated with fatty acids can be identified, this study applied ARS_UCD 1.2 for analysis. Compared with the UMD_3.1, which is used in more studies, this version has been supplemented and updated with more SNP information and is more likely to mine new SNPs that differ from previous findings; for example, Freitas et al. discovered many new SNPs related to milk fatty acids on chromosomes 5, 13, and 14 based on ARS_UCD 1.2 [54]. Therefore, close to our expectations, this study screened and identified 7 novel SNPs significantly associated with fatty acids that had not been previously reported. Bioinformatics analysis indicated that these SNPs might directly or indirectly affect gene expression regulation. *TMEM120B* played a vital role in adipocyte differentiation [89,90]; *CASK* was involved in mammary gland development, milk yield, milk fat percentage, milk protein percentage, and coagulation traits [91,92]; *EVC* and *EYA2* play an essential regulatory role in growth and development of dairy cows [93,94]; *ADORA1* was related to immune system development and regulation [95,96]. Relevant regulatory mechanisms are worth further exploration. Moreover, since cow herds for verification and identification were derived from different pastures with a small sample size, only 7 of the 47 SNPs initially screened in this study were validated to be significant in another cattle herd. Yet, other SNPs were also assumed to be crucial for fatty acid content. Specifically, 8 SNPs were preliminarily selected, showing significant associations with two fatty acids, and 5 of them denoted a strong correlation with carbon chain length or fatty acid type. Meanwhile, Bovine HD 0100019865 was proved to have a significant impact on C10:0 content and a tendency to affect C12:0 content (*p*-value Bonferroni = $8.34\times 10^{-2}$) significantly. Hence, further validation and identification are required for the 47 SNPs selected in this study.

### 4.4. Limitations of the Study

Since cow herds for verification and identification were derived from different pastures with a small sample size, only 7 of the 47 SNPs initially screened in this study were validated to be significant in another cattle herd. Yet, other SNPs were also assumed to be crucial for fatty acid content. Hence, further validation and identification are required for the 47 SNPs selected in this study. In addition, the regulatory mechanisms of fatty acid regulation by the 7 novel SNPs screened in this study merit further exploration. The density of the gene chip is only 50k, and the chip density is low. Only one site is related to the DGAT1 gene, which may lead to missed SNP selection.

Moreover, the accuracy of several models needs to be improved, which could introduce bias in the results. Additionally, the general applicability of the model needs to be verified by methods such as cross-farm and other effect tests. Furthermore, the fatty acid yield in the groups of cows participating in the study was low, meaning that the interactions between the different fatty acids might not be significant enough.

Furthermore, this study was conducted on farms with TMR feeding, and the findings may be more applicable to pastures of the same type rather than to grazing dairy cows.

## 5. Conclusions

This study constructed quantitative prediction models for 14 individual fatty acids and 8 fatty acid groups using FT-MIRS, a D1 or D2 spectral preprocessing algorithm, and a PLSR machine learning algorithm. The model performance was favorable and could be adopted for the quantitative prediction of milk fatty acids. The multi-trait model based on HiBLUP can assess multiple genetic parameters, e.g., individual breeding value, heritability, repeatability, phenotypic correlation, and genetic correlation of dairy cattle. It was shown that Chinese dairy cattle had medium fatty acid heritability and moderate to high repeatability with highly accurate breeding value estimates. Genetic correlations between different fatty acids were predominantly positive. Seven novel SNPs significantly correlated with fatty acid levels were identified using the predicted fatty acid content values from the quantitative prediction models, 50k SNP microarray, DNA sequencing, and superior GWAS algorithm (FarmCPU). The seven SNPs were located in or adjacent to 9 genes that regulate fatty acid synthesis. Also, the prediction model of fatty acid content based on FT-MIRS has high accuracy and versatility. More new SNPs and genes can be identified using higher-density chips or deep resequencing (lower-density chips selected seven significant SNPs) that would be worthy of further probation. The above model and results lay a foundation and provide technical support for detecting milk fatty acid content and investigating genetic characteristics, as well as dairy cow breeding of fatty acid traits. It is hoped that, in the future, the method established in this study will be able to screen milk sources rich in high-quality fatty acids.

## Figures and Tables

**Table 1:** Eight classified fatty acid groups, according to hydrocarbon chain saturation and carbon chain length.

Trait	* Fatty Acids Included
TFA	SFA, UFA/SCFA, MCFA, LCFA
SFA	C4:0, C6:0, C8:0, C10:0, C11:0, C12:0, C13:0, C14:0, C15:0, C16:0, C17:0, C18:0, C20:0, C21:0, C22:0, C23:0, C24:0
UFA	MUFA, PUFA
MUFA	C14:1, C15:1, C16:1, C17:1, C18:1, C20:1, C22:1, C24:1
PUFA	C18:2, C18:3, C20:2, C20:3, C20:4, C20:5, C22:2, C22:6
SCFA	C4:0, C6:0, C8:0, C10:0
MCFA	C11:0, C12:0, C13:0, C14:0, C14:1, C15:0, C15:1,C16:0, C16:1
LCFA	C17:0, C17:1, C18:0, C18:1, C18:2, C18:3, C20:0, C20:1, C20:2, C20:3, C20:4, C20:5, C21:0, C22:0, C22:1, C22:2, C22:6, C23:0, C24:0, C24:1

* TFA = total fatty acids; SFA = saturated fatty acid; UFA = unsaturated fatty acid; MUFA = monounsaturated fatty acids; PUFA = polyunsaturated fatty acids; SCFA = short-chain fatty acids; MCFA = mid-chain fatty acids; LCFA = long-chain fatty acids.

**Table 2:** Descriptive statistics of fatty acid traits.

Trait	^1^ g/100 g of Total Fat					^1^ Percentage of Total Fatty Acid, %				
	^2^ Mean	^2^ SD	^2^ Max	^2^ Min	^2^ CV, %	^2^ Mean	^2^ SD	^2^ Max	^2^ Min	^2^ CV, %
C4:0	1.18	1.06	7.59	0.09	90.55	3.36	3.09	32.44	1.14	92.01
C6:0	0.59	0.32	1.58	0.08	54.22	1.66	0.31	2.32	0.60	18.84
C8:0	0.38	0.21	1.06	0.05	54.38	1.06	0.21	1.54	0.34	19.76
C10:0	1.00	0.57	3.26	0.09	57.47	2.80	0.73	5.02	0.81	26.00
C12:0	1.13	0.65	3.72	0.15	56.97	3.21	0.80	5.88	1.07	24.80
C14:0	3.77	1.95	9.49	0.52	51.56	10.72	1.69	15.22	4.99	15.78
C14:1,cis-9	0.31	0.20	1.21	0.03	62.42	0.89	0.32	2.17	0.20	36.13
C15:0	0.38	0.24	1.69	0.05	61.68	1.08	0.33	2.58	0.38	30.36
C16:0	12.74	6.19	31.06	1.61	48.60	36.37	3.53	49.21	24.89	9.69
C17:0	0.18	0.10	0.62	0.02	55.50	0.51	0.11	0.97	0.27	20.98
C18:0	3.92	2.10	11.50	0.52	53.58	11.15	2.35	22.36	6.19	21.09
C18:1,n9 cis-9	6.53	4.29	30.72	0.05	65.79	17.98	6.73	37.94	0.45	37.42
C18:2,n6cis-9,12	1.00	0.55	2.84	0.12	55.02	2.79	0.55	4.37	1.42	19.56
C18:3,n3cis-9,12,15	0.10	0.07	0.34	0.00	72.92	0.26	0.13	0.88	0.04	50.98
^3^ TFA	35.22	17.14	80.97	4.52	48.67	——	——	——	——	——
^3^ SFA	25.39	12.33	59.80	3.46	48.55	72.30	4.50	90.89	51.57	6.23
^3^ MUFA	8.52	4.65	34.66	0.48	54.65	24.04	4.44	43.55	4.89	18.46
^3^ PUFA	1.33	0.72	3.69	0.15	54.53	3.71	0.68	6.71	2.14	18.42
^3^ UFA	9.85	5.28	37.04	0.89	53.66	27.77	4.52	48.44	9.11	16.26
^3^ SCFA	3.14	1.82	10.14	0.35	58.07	8.88	3.35	37.73	4.72	37.76
^3^ MCFA	18.94	9.32	47.66	2.52	49.20	53.97	5.53	69.22	36.73	10.25
^3^ LCFA	13.14	6.93	44.67	1.62	52.74	37.16	5.79	57.04	22.94	15.58

^1^ The value of fatty acid traits was determined using gas chromatography (GC). ^2^ Mean = mean value; SD = standard deviation; Max = maximum; Min = minimum; CV = coefficient of variation. ^3^ TFA = total fatty acid; SFA = saturated fatty acid; UFA = unsaturated fatty acid; MUFA = monounsaturated fatty acid; PUFA = polyunsaturated fatty acid; SCFA = short-chain fatty acid; MCFA = mid-chain fatty acid; LCFA = long-chain fatty acid.

**Table 3:** Model characteristics and metrics for cross-validation and test sets.

Trait	^1^ Preprocessing Methods	Number of Characteristic Wavelengths	^2^ R_cv_^2^	^2^ RMSE_cv_	^2^ R_t_^2^	^2^ RMSE_t_	^2^ RPD_cv_
C4:0	D2 + D2	585	0.76	0.48	0.65	0.74	2.04
C6:0	D1 + D1	657	0.64	0.19	0.57	0.22	1.67
C8:0	D2 + D2	532	0.76	0.10	0.72	0.12	2.03
C10:0	D1 + D1	647	0.68	0.31	0.65	0.37	1.77
C12:0	D1 + D1	564	0.67	0.36	0.62	0.44	1.74
C14:0	D1 + D1	603	0.64	1.10	0.58	1.44	1.68
C14:1,cis-9	D1 + D1	576	0.67	0.11	0.62	0.14	1.73
C15:0	D1 + D2	688	0.71	0.11	0.60	0.19	1.85
C16:0	D1 + D1	709	0.64	3.71	0.52	4.25	1.67
C17:0	D2 + D2	456	0.64	0.06	0.49	0.07	1.67
C18:0	D1 + D1	510	0.59	1.30	0.60	1.45	1.57
C18:1,n9 cis-9	D1 + D2	561	0.67	2.32	0.58	3.21	1.74
C18:2,n6 cis-9,12	D1 + D1	612	0.65	0.32	0.50	0.39	1.69
C18:3,n3 cis-9,12,15	D1 + D2	693	0.69	0.04	0.44	0.06	1.81
^3^ TFA	D2 + D2	511	0.70	9.24	0.66	10.39	1.82
^3^ SFA	D2 + D2	511	0.71	6.50	0.67	7.52	1.86
^3^ UFA	D2 + D2	469	0.65	3.13	0.65	3.07	1.69
^3^ MUFA	D2 + D2	459	0.65	2.78	0.65	2.68	1.68
^3^ PUFA	D2 + D2	491	0.68	0.40	0.62	0.47	1.77
^3^ SCFA	D2 + D2	561	0.72	0.97	0.74	0.90	1.90
^3^ MCFA	D2 + D2	472	0.66	5.18	0.67	5.88	1.73
^3^ LCFA	D2 + D2	568	0.62	4.12	0.62	4.67	1.62

^1^ D1 = first derivative; D2 = second derivative; D1+D1 = FT-MIRS data were preprocessed twice with D1 employed; D2+D2 = FT-MIRS data were preprocessed twice with D2 employed; D1+D2 = FT-MIRS data were preprocessed once with D1, then with D2. ^2^ R_cv_^2^ = cross-validation coefficient of determination; R_t_^2^ = test set coefficient of determination; RMSE_cv_ = root mean square error in cross-validation; RMSE_t_ = root mean square error in test set; RPD_cv_ = residual predictive deviation of cross-validation. ^3^ TFA = total fatty acid; SFA = saturated fatty acid; UFA = unsaturated fatty acid; MUFA = monounsaturated fatty acid; PUFA = polyunsaturated fatty acid; SCFA = short-chain fatty acid; MCFA = mid-chain fatty acid; LCFA = long-chain fatty acid.

**Table 4:** Variance component estimates and estimated breeding values for FT-MIRS-predicted fatty acid traits.

Fatty Acid	Variance Component Estimates						Estimated Breeding Values			
	^1^ σ_u_^2^	^1^ σ_c_^2^	^1^ σ_e_^2^	^1^ σ_T_^2^	^1^ h^2^	^1^ t	^2^ Mean	^2^ Min	^2^ Max	^2^ Acc
C4:0	0.14	0.20	0.17	0.51	0.28	0.67	−0.03	−0.90	0.89	0.73
C6:0	0.02	0.02	0.03	0.07	0.31	0.59	−0.01	−0.31	0.59	0.71
C8:0	0.01	0.01	0.02	0.03	0.23	0.49	−0.01	−0.18	0.23	0.67
C10:0	0.08	0.06	0.10	0.24	0.33	0.59	−0.02	−0.60	1.10	0.72
C12:0	0.09	0.07	0.11	0.27	0.34	0.59	−0.03	−0.65	0.76	0.73
C14:0	0.54	0.69	0.66	1.89	0.29	0.65	−0.05	−1.95	2.25	0.76
C14:1,cis-9	0.01	0.01	0.02	0.04	0.25	0.48	0.00	−0.20	0.18	0.54
C15:0	0.01	0.01	0.02	0.04	0.29	0.54	0.00	−0.23	0.27	0.70
C16:0	4.01	6.26	4.80	15.06	0.27	0.68	−0.24	−5.73	9.13	0.79
C17:0	0.00	0.00	0.00	0.01	0.20	0.33	0.00	−0.10	0.08	0.63
C18:0	0.43	0.70	0.52	1.65	0.26	0.69	−0.04	−2.00	3.15	0.78
C18:1,n9 cis-9	2.08	2.87	2.48	7.42	0.28	0.67	−0.08	−3.33	3.65	0.69
C18:2,n6 cis-9,12	0.07	0.08	0.09	0.24	0.31	0.62	−0.02	−0.63	0.67	0.70
C18:3,n3 cis-9,12,15	0.00	0.00	0.00	0.00	0.22	0.50	0.00	−0.05	0.06	0.57
^3^ TFA	29.02	35.99	37.39	102.39	0.28	0.64	−0.26	−16.06	26.69	0.82
^3^ SFA	18.91	18.77	24.27	61.94	0.31	0.61	−0.21	−10.20	23.65	0.82
^3^ UFA	4.00	3.97	5.54	13.51	0.30	0.59	−0.11	−5.60	7.00	0.78
^3^ MUFA	3.30	3.27	4.61	11.17	0.30	0.59	−0.10	−5.35	6.79	0.78
^3^ PUFA	0.07	0.07	0.09	0.22	0.30	0.60	−0.01	−0.64	0.79	0.74
^3^ SCFA	0.47	0.65	0.55	1.68	0.28	0.67	−0.06	−1.54	2.45	0.76
^3^ MCFA	8.78	13.79	10.14	32.71	0.27	0.69	−0.20	−8.18	9.77	0.74
^3^ LCFA	5.81	8.78	6.73	21.33	0.27	0.68	−0.19	−7.47	9.66	0.74

^1^ Variance component estimates: σ_u_^2^ = additive genetic variance; σ_c_^2^ = permanent environment variance; σ_e_^2^ = residual variance; σ_T_^2^ = total variance (σ_u_^2^ + σ_c_^2^ + σ_e_^2^); h^2^ = heritability estimate; t = repeatability estimate. ^2^ Estimated breeding values: Using 0.00 as the population mean; Mean = the mean of all individual breeding values; Min = the minimum individual breeding value; Max = the maximum individual breeding value; Acc = the accuracy of breeding value estimation. ^3^ TFA = total fatty acid; SFA = saturated fatty acid; UFA = unsaturated fatty acid; MUFA = monounsaturated fatty acid; PUFA = polyunsaturated fatty acid; SCFA = short-chain fatty acid; MCFA = mid-chain fatty acid; LCFA = long-chain fatty acid.

**Table 5:** Genetic (below the diagonal) and phenotypic (above the diagonal) correlations for FT-MIRS-predicted milk traits.

	Fat	Protein	Lactose	Total Solids	Urea	C4:0	C6:0	C8:0	C10:0	C12:0	C14:0	C14:1, cis-9	C15:0	C16:0	C17:0	C18:0	C18:1,n9 cis-9	C18:2,n6 cis-9,12	C18:3,n3 cis-9,12,15	^1^ TFA	^1^ SFA	^1^ UFA	^1^ MUFA	^1^ PUFA	^1^ SCFA	^1^ MCFA	^1^ LCFA
Fat		0.42	−0.06	0.93	0.08	0.09	0.22	0.18	0.20	0.20	0.14	0.17	0.15	0.27	0.27	0.23	0.18	0.15	0.16	0.19	0.18	0.17	0.18	0.09	0.21	0.20	0.22
Protein	0.66		−0.14	0.66	0.14	−0.07	0.12	0.09	0.21	0.28	0.16	0.22	0.23	0.19	0.11	0.00	−0.16	0.00	0.01	0.03	0.08	−0.04	−0.03	−0.10	0.12	0.23	−0.07
Lactose	0.13	−0.11		0.08	0.05	0.02	−0.23	−0.10	−0.17	−0.18	−0.04	−0.17	−0.03	−0.14	−0.03	−0.08	−0.14	−0.28	−0.22	−0.09	−0.08	−0.10	−0.10	−0.14	−0.11	−0.17	−0.06
Total solids	0.98	0.74	0.18		0.14	0.10	0.21	0.18	0.25	0.26	0.19	0.23	0.21	0.28	0.24	0.20	0.11	0.09	0.13	0.16	0.16	0.12	0.13	0.02	0.23	0.22	0.16
Urea	0.20	0.23	0.10	0.26		0.20	−0.15	0.03	0.02	0.02	−0.02	−0.02	−0.16	−0.12	−0.17	−0.02	−0.10	−0.17	0.01	−0.14	−0.09	−0.14	−0.16	−0.09	0.07	−0.05	−0.21
C4:0	0.21	0.07	−0.02	0.24	0.37		0.09	0.40	0.24	0.14	0.14	0.01	0.01	0.10	0.03	0.33	0.18	0.06	0.25	0.22	0.27	0.13	0.12	0.17	0.55	0.24	0.11
C6:0	0.26	0.12	−0.10	0.28	−0.24	0.07		0.38	0.75	0.76	0.71	0.59	0.64	0.67	0.21	0.50	0.51	0.75	0.57	0.28	0.29	0.17	0.19	0.21	0.27	0.41	0.41
C8:0	0.32	0.23	−0.16	0.35	0.06	0.68	0.43		0.46	0.42	0.38	0.24	0.32	0.35	0.21	0.46	0.28	0.30	0.40	0.46	0.50	0.29	0.28	0.37	0.59	0.45	0.25
C10:0	0.30	0.24	−0.09	0.34	−0.05	0.29	0.87	0.60		0.87	0.72	0.63	0.54	0.72	0.10	0.50	0.40	0.59	0.65	0.33	0.39	0.17	0.17	0.31	0.46	0.54	0.30
C12:0	0.26	0.28	−0.06	0.32	−0.05	0.16	0.86	0.51	0.94		0.75	0.64	0.54	0.70	0.12	0.43	0.31	0.58	0.55	0.26	0.32	0.11	0.10	0.24	0.32	0.50	0.22
C14:0	0.28	0.24	−0.02	0.34	−0.10	0.27	0.88	0.61	0.93	0.92		0.61	0.70	0.61	0.11	0.48	0.36	0.55	0.54	0.31	0.35	0.16	0.16	0.19	0.32	0.51	0.32
C14:1,cis−9	0.25	0.20	−0.10	0.28	−0.06	−0.01	0.80	0.35	0.82	0.84	0.81		0.60	0.65	0.15	0.31	0.42	0.55	0.43	0.27	0.27	0.20	0.21	0.16	0.22	0.39	0.31
C15:0	0.22	0.28	0.01	0.26	−0.24	0.10	0.65	0.42	0.64	0.66	0.80	0.65		0.53	0.27	0.31	0.40	0.56	0.43	0.37	0.37	0.23	0.24	0.21	0.25	0.56	0.43
C16:0	0.40	0.21	−0.04	0.42	−0.27	0.24	0.89	0.61	0.88	0.84	0.94	0.79	0.79		0.28	0.47	0.42	0.60	0.52	0.37	0.38	0.25	0.25	0.29	0.29	0.45	0.41
C17:0	0.32	0.15	−0.03	0.31	−0.25	0.16	0.30	0.34	0.26	0.25	0.33	0.32	0.38	0.49		0.27	0.19	0.22	0.09	0.68	0.61	0.61	0.60	0.51	0.21	0.23	0.37
C18:0	0.38	0.09	0.01	0.39	0.00	0.50	0.74	0.68	0.74	0.68	0.77	0.66	0.51	0.82	0.53		0.41	0.45	0.61	0.44	0.44	0.41	0.41	0.35	0.44	0.35	0.35
C18:1,n9 cis-9	0.28	−0.11	0.03	0.26	−0.13	0.15	0.58	0.28	0.43	0.35	0.55	0.51	0.58	0.69	0.29	0.56		0.66	0.54	0.27	0.19	0.34	0.34	0.27	0.20	0.31	0.60
C18:2,n6 cis-9,12	0.17	−0.04	−0.11	0.17	−0.25	0.04	0.86	0.35	0.70	0.68	0.78	0.79	0.68	0.83	0.24	0.68	0.80		0.61	0.25	0.20	0.21	0.21	0.30	0.20	0.37	0.51
C18:3,n3 cis-9,12,15	0.27	0.03	−0.16	0.29	−0.02	0.40	0.77	0.69	0.84	0.72	0.85	0.75	0.58	0.87	0.34	0.82	0.70	0.82		0.35	0.38	0.26	0.25	0.41	0.42	0.48	0.41
^1^ TFA	0.26	0.11	−0.09	0.26	−0.15	0.41	0.38	0.68	0.47	0.39	0.52	0.33	0.47	0.63	0.85	0.66	0.35	0.30	0.57		0.97	0.85	0.84	0.80	0.49	0.48	0.38
^1^ SFA	0.25	0.13	−0.09	0.26	−0.13	0.42	0.36	0.68	0.47	0.39	0.51	0.31	0.45	0.60	0.84	0.65	0.29	0.26	0.55	1.00		0.75	0.73	0.77	0.56	0.52	0.29
^1^ UFA	0.18	−0.05	−0.06	0.15	−0.15	0.28	0.22	0.49	0.24	0.16	0.29	0.20	0.28	0.44	0.87	0.56	0.34	0.21	0.42	0.94	0.93		1.00	0.75	0.29	0.33	0.41
^1^ MUFA	0.17	−0.07	−0.06	0.14	−0.16	0.26	0.21	0.47	0.22	0.14	0.27	0.20	0.27	0.43	0.87	0.56	0.35	0.21	0.41	0.93	0.92	1.00		0.71	0.29	0.32	0.42
^1^ PUFA	0.11	−0.05	−0.14	0.11	−0.11	0.31	0.24	0.54	0.36	0.27	0.37	0.21	0.32	0.47	0.77	0.49	0.28	0.19	0.49	0.92	0.92	0.91	0.89		0.39	0.41	0.38
^1^ SCFA	0.33	0.26	−0.09	0.37	0.11	0.81	0.31	0.85	0.52	0.40	0.55	0.19	0.43	0.55	0.48	0.63	0.23	0.20	0.59	0.75	0.76	0.55	0.52	0.62		0.47	0.26
^1^ MCFA	0.35	0.25	−0.07	0.38	−0.15	0.47	0.58	0.74	0.68	0.59	0.78	0.46	0.77	0.82	0.56	0.64	0.58	0.55	0.76	0.79	0.78	0.59	0.56	0.67	0.82		0.44
^1^ LCFA	0.31	0.01	−0.01	0.29	−0.30	0.19	0.56	0.42	0.46	0.37	0.61	0.44	0.71	0.76	0.44	0.53	0.92	0.73	0.70	0.55	0.49	0.47	0.47	0.45	0.43	0.80	

^1^ TFA = total fatty acid; SFA = saturated fatty acid; UFA = unsaturated fatty acid; MUFA = monounsaturated fatty acid; PUFA = polyunsaturated fatty acid; SCFA = short-chain fatty acid; MCFA = mid-chain fatty acid; LCFA = long-chain fatty acid. Correlations between fatty acids and conventional milk components: There was a low to moderate positive correlation between 22 fatty acids and milk fat or total solids (0.11–0.42). Milk protein was positively associated with short- or medium-chain fatty acids (0.07–0.28), weakly positively or negatively correlated with long-chain fatty acids, weakly negatively correlated with fatty acid groups UFA, MUFA, and PUFA, and moderately positively related to SCFA and MCFA. Except for urea nitrogen and C4:0 (0.37), lactose or urea nitrogen showed a weak positive correlation or weak to low negative correlation (−0.30–0.11) with fatty acids. To conclude, milk fat/total solids and fatty acids had congruous variation trends, while lactose/urea nitrogen had an opposite variation trend to fatty acids. Milk protein displayed a consistent variation trend with short- or medium-chain fatty acids, showing little correlation with long-chain or unsaturated fatty acids.

**Table 6:** SNP loci significantly related to fatty acid content screened by the FarmCPU method.

Name	SNP	Chrom	Position	Genetic Variation	Effect	Raw *p*-Value	*p*-Value Bonferroni	Gene	Functional Region
C4:0	BTB-01865288	6	g.39109137	A > C	−0.091298053	$1.08\times 10^{-6}$	0.873	LCORL (dist = 1,552,031), SLIT2 (dist = 670,758)	Intergenic
C4:0	BovineHD2200013291	22	g.45609641	T > C	0.109639913	$1.33\times 10^{-8}$	0.114	WNT5A (dist = 51,524), CACNA2D3 (dist = 314,894)	Intergenic
C6:0	BovineHD0100039519	1	g.156602608	T > G	0.034016364	$6.84\times 10^{-7}$	0.141	KCNH8	Intronic
C6:0	BovineHD1400023113	14	g.79488283	A > G	0.04046719	$6.23\times 10^{-7}$	0.439	ENSBTAG00000046821 (dist = 838,481), \SNX16 (dist = 1,274,216)	Intergenic
C6:0	ARS-BFGL-NGS-22276	17	g.53567893	A > G	0.03801704	$7.02\times 10^{-8}$	$8.80\times 10^{-3}$	TMEM120B	Upstream
C6:0	BovineHD2100014553	21	g.50065194	A > G	0.056103731	$8.20\times 10^{-9}$	0.607	FBXO33 (dist = 755,051), LRFN5 (dist = 1,263,846)	Intergenic
C8:0	BovineHD0100039519	1	g.156602608	T > G	0.018626629	$4.76\times 10^{-8}$	0.758	KCNH8	Intronic
C8:0	ARS-BFGL-NGS-33001	6	g.103480813	A > G	−0.017966404	$4.94\times 10^{-7}$	$3.02\times 10^{-2}$	EVC	Exonic
C8:0	BovineHD0800031399	8	g.103616676	T > G	0.021357656	$1.88\times 10^{-8}$	0.892	AKNA (dist = 11,743), WHRN (dist = 4323)	Intergenic
C8:0	BovineHD1200017499	12	g.63379103	A > C	0.018193801	$9.07\times 10^{-8}$	0.104	ENSBTAG00000051320 (dist = 61,739), NONE (dist = NONE)	Intergenic
C8:0	BovineHD2900002136	29	g.7493345	A > C	−0.0197566	$1.96\times 10^{-8}$	0.146	CTSC (dist = 76,111), RAB38 (dist = 7945)	Intergenic
C10:0	BovineHD0100019865	1	g.69045145	T > C	−0.053724787	$5.00\times 10^{-7}$	$9.66\times 10^{-3}$	ENSBTAG00000053238	Intronic
C10:0	Hapmap48014-BTA-59805	5	g.15293572	T > C	0.048419799	$4.36\times 10^{-7}$	0.413	ALX1 (dist = 354,357), RASSF9 (dist = 174,821)	Intergenic
C10:0	BovineHD2900009379	29	g.30870766	A > G	−0.053948553	$1.96\times 10^{-7}$	0.793	ENSBTAG00000052770 (dist = 293,780), ETS1 (dist = 942,420)	Intergenic
C10:0	BovineHD3000029498	X	g.102101013	A > G	−0.05687289	$4.30\times 10^{-7}$	0.971	CASK (dist = 28,036), NYX (dist = 6763)	Intergenic
C12:0	BovineHD0100019865	1	g.69045145	T > C	−0.067241259	$2.27\times 10^{-7}$	$8.34\times 10^{-2}$	ENSBTAG00000053238	Intronic
C14:0	BovineHD1500018842	15	g.64979528	T > C	0.202541955	$1.07\times 10^{-7}$	0.997	CAT	Intronic
C14:0	BovineHD3000029498	X	g.102101013	A > G	−0.199760831	$1.07\times 10^{-6}$	$3.56\times 10^{-2}$	CASK (dist = 28,036), NYX (dist = 6763)	Intergenic
C14:1,cis-9	BovineHD1300014447	13	g.49614540	T > C	0.023699371	$1.28\times 10^{-7}$	0.351	BMP2 (dist = 428,961), ENSBTAG00000007199 (dist = 932,496)	Intergenic
C14:1,cis-9	ARS-BFGL-NGS-75890	14	g.65908412	A > G	0.558449636	$9.26\times 10^{-7}$	—	——	——
C14:1,cis-9	BovineHD1600000152	16	g.896630	T > C	−0.019590243	$1.13\times 10^{-6}$	$1.57\times 10^{-7}$	ENSBTAG00000050274 (dist = 75,663), ADORA1 (dist = 18,476)	Intergenic
C14:1,cis-9	Hapmap27983-BTA-163473	16	g.17975275	T > C	0.194892692	$2.79\times 10^{-7}$	——	——	——
C14:1,cis-9	BovineHD3000036571	X	g.121649547	T > C	0.012453536	$4.56\times 10^{-7}$	0.781	CNKSR2	Intronic
C15:0	BovineHD0400022351	4	g.80194206	A > C	0.04267447	$4.33\times 10^{-9}$	0.801	——	——
C15:0	BovineHD2200010872	22	g.38049578	A > G	0.025775523	$8.15\times 10^{-8}$	0.392	SYNPR	Intronic
C15:0	BovineHD3000019134	X	g.60227886	T > C	0.024999374	$5.98\times 10^{-7}$	0.124	TRPC5	Intronic
C16:0	BovineHD0400022351	4	g.80194206	A > C	0.04267447	$4.33\times 10^{-9}$	0.650	——	——
C16:0	BovineHD2200010872	22	g.38049578	A > G	0.025775523	$8.15\times 10^{-8}$	0.771	SYNPR	Intronic
C16:0	BovineHD3000019134	X	g.60227886	T > C	0.024999374	$5.98\times 10^{-7}$	0.984	TRPC5	Intronic
C18:0	BovineHD2200008234	22	g.28238616	A > G	0.268793511	$7.04\times 10^{-7}$	0.247	——	——
C18:0	BovineHD3000036571	X	g.121649547	T > C	0.217017144	$9.08\times 10^{-7}$	0.397	CNKSR2	intronic
C18:1,n9 cis-9	ARS-BFGL-NGS-100563	5	g.98601044	T > G	0.663309395	$1.24\times 10^{-8}$	0.761	ENSBTAG00000001336 (dist = 4735), ENSBTAG00000054460 (dist = 18,399)	Intergenic
C18:1,n9 cis-9	ARS-USDA-AGIL-chr26-44746016-000548	26	g.44408705	T > G	0.552863575	$1.13\times 10^{-6}$	0.902	——	——
C18:1,n9 cis-9	BTB-01892890	29	g.6982262	T > C	0.332464096	$3.06\times 10^{-7}$	0.374	TYR (dist = 546,774), GRM5 (dist = 25,615)	Intergenic
C18:2,n6 cis-9,12	BTB-00387033	9	g.28842247	T > C	0.071584274	$5.81\times 10^{-7}$	0.454	HSF2 (dist = 39,233), ENSBTAG00000053171 (dist = 266,891)	Intergenic
C18:2,n6 cis-9,12	BovineHD1200006970	12	g.23185693	T > C	−0.102781399	$5.56\times 10^{-7}$	0.839	——	——
C18:2,n6 cis-9,12	BovineHD3000011570	X	g.35028719	A > G	0.079096988	$2.01\times 10^{-7}$	0.230	ENSBTAG00000048998 (dist = 65,578), GABRQ (dist = 17,494)	Intergenic
C18:3,n3 cis-9,12,15	ARS-BFGL-NGS-34990	8	g.11469311	T > C	0.006830786	$9.84\times 10^{-7}$	0.754	TMEM215 (dist = 133,759), NDUFB6 (dist = 35,314)	Intergenic
C18:3,n3 cis-9,12,15	AX-27555724	8	g.83221868	T > G	−0.006388075	$1.11\times 10^{-6}$	0.714	CDC14B	Exonic
C18:3,n3 cis-9,12,15	ARS-BFGL-NGS-15402	17	g.11368599	T > C	0.007664341	$1.66\times 10^{-7}$	$8.34\times 10^{-3}$	TTC29	Intronic
^1^ UFA	ARS-BFGL-NGS-27018	X	g.29070096	T > C	0.671700039	$3.75\times 10^{-7}$	0.293	——	——
^1^ MUFA	ARS-BFGL-NGS-27018	X	g.29070096	T > C	0.671700039	$3.75\times 10^{-7}$	0.330	——	——
^1^ SCFA	BovineHD0300028970	3	g.100601230	A > G	−0.185365212	$1.72\times 10^{-7}$	0.465	TESK2	Intronic
SCFA	BovineHD0700028128	7	g.94179851	A > G	−0.27438426	$1.39\times 10^{-7}$	0.530	MCTP1	Intronic
^1^ LCFA	BovineHD1300022078	13	g.75585136	A > G	0.580890257	$4.50\times 10^{-7}$	$4.61\times 10^{-3}$	EYA2	Intronic
LCFA	ARS-USDA-AGIL-chr24-46907845-000534	24	g.46441847	A > C	−0.961849131	$2.07\times 10^{-10}$	0.267	ST8SIA5	Intronic
LCFA	ARS-BFGL-NGS-93595	24	g.52964581	A > G	2.190506381	$1.76\times 10^{-7}$	0.197	——	——

^1^ UFA = unsaturated fatty acid; MUFA = monounsaturated fatty acid; SCFA = short-chain fatty acid; LCFA = long-chain fatty acid. Subsequently, 47 SNPs from the above preliminary selected SNPs were genotyped in 265 Chinese Holstein cows from another dairy herd by DNA sequencing and were analyzed and validated by Bonferroni ANOVA. Seven important SNPs related to fatty acid content were further identified from the forty-seven SNPs. The specific results are as follows: Single short-chain fatty acids: 2, 4, 5, and 4 SNPs were found to be significantly associated with C4:0, C6:0, C8:0, and C10:0, respectively. These 15 SNPs were distributed on 11 chromosomes, of which 10 were located between genes, 3 in introns, 1 in exon, and 1 in the upstream of a gene. BovineHD0100039519 was significantly correlated with C6:0 and C8:0 and located in the intron of the *KCNH8*. Three SNPs, ARS-BFGL-NGS-22276 (C6:0, *p*-value Bonferroni = $8.80\times 10^{-3}$), ARS-BFGL-NGS-33001 (C8:0, *p*-value Bonferroni = $3.02\times 10^{-2}$), and Bovine HD 0100019865(C10:0, *p*-value Bonferroni = $9.66\times 10^{-3}$), were identified having significant effects on fatty acid content from 15 SNPs. The three SNPs were located upstream of the *TMEM120B*, the exon of the *EVC*, and the intron of the *ENSBTAG00000053238*, respectively.

## Data Availability

The datasets used and/or analyzed during the current study are available from the corresponding authors on reasonable request.

## Supplementary Materials

The following supporting information can be downloaded at: https://www.mdpi.com/article/10.3390/ani14192901/s1, Table S1: Distribution of samples; Folder S1: Corrected phenotypes.
